# Relation between sexual function, perceived social support, and adherence to treatment with infertility factor in women: A cross-sectional study

**DOI:** 10.18502/ijrm.v21i7.13893

**Published:** 2023-08-23

**Authors:** Marzie Sheikhian, Marzeyeh Loripoor, Zohreh Ghorashi, Faranak Safdari-Dehcheshmeh

**Affiliations:** ^1^Department of Midwifery, School of Nursing and Midwifery, Rafsanjan University of Medical Sciences, Rafsanjan, Iran.; ^2^Social Determinants of Health Research Center, Shahrekord University of Medical Sciences, Shahrekord, Iran.; ^3^Department of Midwifery and Reproductive Health, School of Nursing and Midwifery, Shahrekord University of Medical Sciences, Shahrekord, Iran.

**Keywords:** Infertility, Sexual health, Social support, Treatment adherence.

## Abstract

**Background:**

In some societies, childbearing is a part of women's identity and infertile women are under a great amount of pressure from others.

**Objective:**

This study aims to evaluate the relationship between infertility factors and sexual functioning, perceived social support, and adherence to treatment in infertile women.

**Materials and Methods:**

In this cross-sectional study, 230 infertile women who referred to the infertility center of Shahrekord, Iran in 2022 were enrolled. Data were collected using demographic characteristics checklist, female sexual function index, multidimensional scale of perceived social support, and general adherence scale.

**Results:**

No significant relation was observed between the infertility factor and the mean score of sexual function, the mean score of perceived social support, and the mean score of adherence to treatment (p > 0.05). Among the aspects of sexual functioning, only the mean score of lubrication in the group of male factors was significantly higher than the common factors for men and women (p = 0.07). A linear positive relation was observed between sexual functioning (r = 0.189), perceived social support (r = 0.200), and adherence to treatment (r = 0.146) in infertile women.

**Conclusion:**

By providing proper training and counseling to infertile couples, we can improve their social support and sexual function so that they can complete their infertility treatment as a result.

## 1. Introduction

Globally, about 10% of the population and 15% of couples during their fertility ages, suffer infertility (1-4). The prevalence of primary infertility in Iran in 2019 was 20.2% (5). Infertility could be stressful for couples, but the experience of this stress is different (3). Women may be blamed by their husbands and families, and even because of a woman's inability to have children, her husband may remarry, and this issue can be extremely stressful for an infertile woman (6). In pronatalist societies, motherhood is the determinative element of being a woman (7). Infertility as a “social disorder” would not only threaten the family dreams of a woman but would also threaten her own feelings (8). Some societies, like Iran, believe that childbearing is one of the most prominent features of women; therefore, when fertility problems occur, a great amount of pressure would be upon women from others such as spouse, family, and friends (9). However, it has not been determined whether a woman's infertility as an infertility factor is associated with sexual function, perceived social support, and adherence to treatment or not.

Since infertile couples are concerned about having a child during their intercourse, the concern that they would face another failure would increase their stress and interfere with their sexual function (10). In a study, 93.9% of the individuals with unexplained infertility and 89.6% of the participants with polycystic ovarian syndrome had sexual dysfunction (11). Another study showed no significant difference between the sexual function in fertile and infertile women (12). Results of the other study showed that the rate of marital satisfaction was higher in women with male infertility factors in comparison to women who were infertile themselves (13).

It has been observed that infertile women receive less social support in comparison to fertile women (14). Results of one study revealed that women who were infertile themselves had received less social support in comparison to women who had infertile husbands (15).

Sexual dysfunction, depression, anxiety, disappointment, the feeling of guilt and worthlessness caused by infertility, and the economic problems of infertility might affect adherence to treatment in infertile women (16, 17). In some studies, treatment adherence was undesirable in infertile women (18); while in the systematic review study adherence to treatment has been reported as 26-81% in infertile women (19).

In societies that consider childbearing as a pillar and part of the women's identity based on their cultural norms, and having a child is considered as the source of power for women in the family and society (20), it seems that female infertility factor might be associated with sexual functionality, perceived social support, and adherence to treatment in infertile women.

This study aimed to investigate the relationship between infertility factors and sexual function, perceived social support, and adherence to treatment in infertile women.

## 2. Materials and Methods

The present descriptive cross-sectional study was conducted from January to March 2022 in the infertility center of Shahrekord, Iran. Based on the study by Direkvand-Moghadam (21) and using the intended statistical formulas and considering a 10% loss of the samples, 230 eligible women were enrolled in the study. Participants aged between 15 and 49 yr, having been diagnosed with infertility, willing to participate in the study, able to read and write, not having a history of any known mental disorder or using drugs for treating mental disorders, not having a history of severe mental pressure during the past 3 months such as an accident or losing a first degree relative, and not being hospitalized recently due to Covid-19, were included in the study; participants were excluded from the study in case of not completing the questionnaires.

Data were collected using demographic characteristics checklist for both husband and wife, which included age, educational level, occupation, place of residence, ethnicity, monthly income of the family, costs of infertility treatments, insurance coverage for infertility treatments, method of contraception, duration of marital life, history of divorce, duration of infertility, type of infertility, cause of infertility, number of children in case of secondary infertility and medical history. Female sexual function index (FSFI), multidimensional scale of perceived social support (MSPSS), and general adherence scale (GAS) were also completed by the participants.

FSFI contains 19 questions, developed by Rosen, Brown, and Heiman in 2000. It evaluates women's sexual functionality during the past 4 wk in 6 independent aspects of sexual desire, arousal, lubrication, orgasm, satisfaction, and sexual pain. The total score of the questionnaire would be achieved by summing up the scores of all the aspects, and higher scores indicate better sexual functionality. By equalizing the aspects, the minimum and maximum scores for the questionnaire are 2 and 36 respectively. The maximum score for each aspect was 6 and the minimum score of the sexual desire aspect was 1.2; the arousal, lubrication, orgasm, and sexual pain aspects were 0; and the satisfaction aspect was 0.8. A total score of sexual functionalities > 26.55 is considered as sexual dysfunction (22). In a study conducted in Iran, the Cronbach's α of this questionnaire was 0.983 using the test-retest method (23).

MSPSS is a 12-item scale and evaluates social support from 3 sources such as family, community, and friends ranging from 1 (totally disagreed) to 7 (totally agreed). The minimum and maximum scores of the scale are 12 and 84 and 4 and 28 respectively, for each of the subscales of family, community, and friends. Higher scores indicate higher perceived social support. Psychometric characteristics of MSPSS have been approved by national and international studies (24, 25). Also, Cronbach's α for this scale has been reported as 0.87, 0.91, and 0.89 in some studies (24, 26, 27).

GAS was developed by Hays in 1994 and evaluates the desire of participants to follow the physician's orders and contains 5 items scored using a 6-point Likert scale. The lowest score on this scale is 6 and the highest score is 30. Higher scores indicate more adherence to treatment. In the study of Hays, the validity of the scale was approved with an acceptable internal consistency (R = 0.81) using structure validity. The Cronbach's α of the scale was 0.6 using the test-retest method (28). The Cronbach's α of this scale was reported as 0.66 in conducted national studies (29).

### Ethical considerations

This study is approved by the Research Ethics Committees of Rafsanjan University of Medical Sciences, Rafsanjan, Iran (Code: IR.RUMS.REC.1400.221). All individuals participated in the study after signing the informed consent form.

### Statistical analysis

Data were analyzed using Version 18 SPSS (SPSS Inc., Chicago, IL) and independent *t* test, one-way variance analysis, Tukey's post hoc test, and Pearson correlation coefficient. A p-value less than 0.05 was considered significant.

## 3. Results

In the present study of 253 studied infertile women, 10 were not willing to participate in the study and 13 were excluded from the study because of their incomplete questionnaires. Eventually, 230 infertile women, with the mean age of 23.37 ± 6.32 yr and a marital duration of 95.53 ± 68.89 months who mostly had primary infertility (144, 62.6%) were enrolled in the study. 27 participants were infertile due to male infertility factor (11.7%), 95 (41.3%) had female infertility, 73 (31.7%) had both female and male infertility, and 35 participants (15.2%) had unexplained infertility. Among the participants, the most common male and female factors were varicocele (39%) and polycystic ovarian syndrome (51.8%), respectively (Table I).

Results of the study showed that the mean scores of sexual functions, perceived social support, and adherence to treatment in infertile women were 22.35 ± 7.07, 57.07 ± 16.01, and 23.31 ± 4.69, respectively. According to the results, 174 participants (75.7%) had sexual dysfunction and 56 (24.3%) did not have sexual dysfunction; meaning that most of the participating women in the study had sexual dysfunction (Table II).

The mean score of lubrication was significantly higher in the male factor group in comparison to both male and female factors (p = 0.03); however, no significant difference was observed between the groups (p > 0.05) in other aspects of sexual function. Also, the mean scores of perceived social support and adherence to treatment were not statistically different between the groups of infertility factors (p > 0.05) (Table III).

Results also showed a linear positive relationship between sexual function, perceived social support, and adherence to treatment in infertile women in a way that improved social support led to better sexual function and more adherence to treatment (Table IV).

**Table 1:** Demographic characteristics of the participated infertile women


**Variables**		**Average of demographic characteristics Mean ± SD/n (%)**
Age (yr)*		
	**Woman**	23.37 ± 6.32
	**Husband**	36.58 ± 7.40
Duration of marriage (months) ${}^{*}$		95.53 ± 63.89
Costs of infertility treatment ${}^{**}$		
	**< 2 millions**	36 (15.7)
	**2-5 millions**	45 (19.5)
	**5-10 millions**	48 (20.9)
	**> 10 millions**	101 (43.9)
	**Total number of variables**	230 (100)
Insurance coverage ${}^{**}$		
	**Lack of insurance coverage**	78 (33.9)
	**Total insurance coverage**	4 (1.7)
	**Partial insurance coverage**	148 (64.4)
	**Total number of variables**	230 (100)
Duration of infertility ${}^{**}$		
	**< 1 yr**	26 (11.3)
	**1-5 yr**	125 (54.3)
	**5-10 yr**	42 (18.3)
	**> 10 yr**	37 (16.1)
	**Total number of variables**	230 (100)
Cause of infertility ${}^{**}$		
	**Male**	27 (11.7)
	**Female**	95 (41.3)
	**Mixed (male and female)**	73 (31.7)
	**Unexplained **	35 (15.3)
	**Total number of variables**	230 (100)
Male factor ${}^{**}$		
	**Azoospermia**	27 (27)
	**Oligospermia**	14 (14)
	**Varicocele**	39 (39)
	**Other causes**	20 (20)
	**Total number of variables**	100 (100)
Female factor ${}^{**}$		
	**Polycystic ovarian syndrome**	87 (51.8)
	**Blocked fallopian tubes**	19 (11.3)
	**Uterine anomaly**	6 (3.6)
	**Endometriosis**	25 (14.8)
	**Asherman syndrome**	8 (4.8)
	**Other causes**	23 (13.7)
	**Total number of variables**	168 (100)
Type of infertility ${}^{**}$		
	**Primary**	144 (62.6)
	**Secondary**	86 (37.4)
	**Total number of variables**	230 (100)
Infertility treatment ${}^{**}$		
	**No**	29 (12.6)
	**Yes**	201 (87.4)
	**Total number of variables**	230 (100)
Type of infertility treatment ${}^{**}$		
	**Medicinal**	85 (42.3)
	**Medicinal and surgical**	88 (43.7)
	**Other methods**	28 (14)
	**Total number of variables**	201 (100)
Duration of infertility treatment ${}^{**}$		
	**< 1 yr**	44 (21.9)
	**1-5 yr**	108 (53.7)
	**> 5 yr**	49 (24.4)
	**Total number of variables**	201 (100)
*Data presented as Mean ± SD. **Data presented as n (%)		

**Table 2:** Mean score and standard deviation of sexual function, perceived social support, and adherence to treatment


**Variables**		**Mean ± SD/n (%)**
Sexual function ${}^{*}$		
	**Sexual desire**	3.45 ± 0.72
	**Arousal**	3.24 ± 1.30
	**Lubrication**	3.73 ± 1.47
	**Orgasm**	3.82 ± 1.61
	**Satisfaction**	4.12 ± 1.53
	**Pain**	3.97 ± 1.72
Total score of FSF*		22.35 ± 7.07
Sexual dysfunction ${}^{**}$		
	**Sexual dysfunction (score < 26.55)**	174 (75.7)
	**Lack of sexual dysfunction (score > 26.55)**	56 (24.3)
	**Total number of variables**	230 (100)
Perceived social support ${}^{*}$		
	**Family support**	20.39 ± 5.86
	**Community support**	20.45 ± 6.36
	**Friends support**	16.23 ± 6.84
Total score of MSPSS*		57.07 ± 16.01
Adherence to treatment ${}^{*}$		
	Total score of GAS*	23.31 ± 4.69
*Data presented as Mean ± SD. **Data presented as n (%). FSFI: Female sexual function index, MSPSS: Multidimensional scale of perceived social support, GAS: General adherence scale		

**Table 3:** Comparing the mean scores of sexual function, perceived social support, and adherence to treatment between various groups of infertility factors


**Variables**/**Type of infertility**		**Male**	**Female**	**Mixed**	**Unexplained**	**P-value**
**Sexual function**						
	**Sexual desire**	3.51 ± 0.51	3.46 ± 0.77	3.46 ± 0.77	3.34 ± 0.75	0.76
	**Arousal**	3.59 ± 1.12	3.41 ± 1.35	2.97 ± 1.30	3.09 ± 1.46	0.06
	**Lubrication**	4.33 ± 1.20	3.87 ± 1.30	3.45 ± 1.60	3.47 ± 1.66	0.02*
	**Orgasm**	4.44 ± 1.43	3.91 ± 1.41	3.59 ± 1.72	3.60 ± 1.87	0.08
	**Satisfaction**	4.71 ± 1.26	4.12 ± 1.49	4.02 ± 1.55	3.91 ± 1.75	0.17
	**Pain**	4.43 ± 1.42	4.11 ± 1.66	3.73 ± 1.75	3.76 ± 1.97	0.20
**Total score of FSFI**		25.04 ± 5.81	22.89 ± 6.52	21.21 ± 7.33	21.17 ± 8.27	0.06
**Perceived social support**						
	**Family support**	21.85 ± 5.45	19.27 ± 5.99	20.71 ± 5.91	21.63 ± 5.35	0.07
	**Community support**	21.19 ± 7.16	86.19 ± 6.38	20.59 ± 6.27	21.17 ± 5.95	0.65
	**Friends support**	15.59 ± 6.96	15.29 ± 6.61	16.42 ± 7.62	18.86 ± 5.00	0.06
	**Total score of MSPSS **	58.63 ± 15.51	54.43 ± 15.72	57.73 ± 17.00	61.66 ± 14.22	0.11
**Adherence to treatment**						
	**Total score of GAS**	24.78 ± 4.59	23.06 ± 4.64	23.18 ± 4.60	23.14 ± 5.05	0.39
Data presented as Mean ± SD. *P < 0.05, One-way variance analysis, FSFI: Female sexual function index, MSPSS: Multidimensional scale of perceived social support, GAS: General adherence scale						

**Table 4:** The correlation coefficient between sexual function, perceived social support, and adherence to treatment


**Total score**	**1**	**2**	**3**
**FSFI**	- -	-
**MSPSS**	r = 0.189, *p < 0.001	- -
**GAS**	r = 0.200, *p < 0.001	r = 0.146, *p = 0.02	-
*P < 0.05, Pearson correlation coefficient. FSFI: Female sexual function index, MSPSS: Multidimensional scale of perceived social support, GAS: General adherence scale			

## 4. Discussion

The present study evaluated the relationship between infertility factors and sexual function, perceived social support, and adherence to treatment in infertile women.

Infertility includes types of female, male, mixed, and unexplained infertility, in which the female infertility is the most common factor. In the present study, the most prevalent type of infertility was female infertility (41.3%) and among the female infertility factors, polycystic ovarian syndrome (51.8%) was the most common factor, which was in line with the results of most of the conducted studies (1, 7, 30, 31). But in one study, the most prevalent factor of infertility reported was male infertility, which was different from the results of the present study (32). The difference in these results might be due to the climatic differences between the studied areas and their cultural and social characteristics. Among the studied participants the most common cause of male infertility was varicocele. In a similar study, varicocele was reported to be the most common cause of male infertility (30). However, some other studies have mentioned oligospermia disorder and impaired sperm motility as the most common cause of male infertility (1, 31, 32). In these studies, only spermogram was evaluated to investigate male infertility factors while in the present study, other causes of infertility such as anatomical causes like varicocele were also investigated, and therefore, the results of the present study varies from the results of those previous studies.

Infertility can affect different aspects of the life of infertile women, so studies have shown that infertile women often suffer from sexual dysfunction and receive unfavorable social support from their spouses, family, and acquaintances. On the other hand, because they consider infertility treatments to be the only way to solve their problem, they follow the optimal treatment. Results of the present study showed that 174 participants (75.7%) had sexual dysfunction and 56 participants (24.3%) had no sexual dysfunction, meaning that most of the infertile women are suffering from sexual dysfunction. In one study 93.9% of the individuals with an unexplained infertility and 89.6% of the participants with polycystic ovarian syndrome, had sexual dysfunction (11). But in the other study 31.2% of infertile women had sexual dysfunction and most of them had desirable sexual functionality (22). The difference between the results of this study and the recent study might be due to the differences in study design such as having a control group and smaller sample size in the mentioned study and also cultural differences, since the sampling of these studies has been conducted in different countries.

The mean score of perceived social support and adherence to treatment in the present study was similar to most of the previously conducted studies and had a desirable level (19, 33). In one study, the perceived social support by infertile women who participated in the study was low and undesirable (34). The participants in the mentioned study underwent IVF treatment, while in our study, the female participants were receiving various infertility treatments. In the other study, which evaluated the effective factors on adherence to treatment in infertile women suffering from polycystic ovarian syndrome, it was revealed that 25.6% of the participants had a desirable level of adherence to treatment (18). In the mentioned study, only women suffering from polycystic ovarian syndrome were studied; the sample size was smaller, and the questionnaires were also different.

Regarding the relationship between sexual function and the factor of infertility, contradictory results have been stated so that in some studies, sexual dysfunction is observed more with the presence of male factor of infertility, while in some, sexual dysfunction is observed more with the presence of female factor of infertility. In some studies, no relationship between the factor of infertility and sexual function has been observed. According to the results, among the aspects of sexual function, a significant relation was observed only between lubrication and infertility factor in a way that the mean score of lubrication was significantly higher in the group with male infertility in comparison to the group with both male and female infertility. Also, in a similar study, no significant relation was observed between the mean of women's sexual functionality and its aspect with infertility factors (11). However, in the other study, the sexual functionality of infertile women had a lower level than infertile men (35). In one study, which evaluated sexual functionality in infertile women suffering from polycystic ovarian syndrome, no significant relation was observed between sexual arousal, orgasm, and satisfaction with infertility factors; but the score of sexual desire was significantly higher in women suffering from polycystic ovarian syndrome and also the score of sexual pain was significantly higher in the group suffering from unexplained infertility (36). This contradiction between the mentioned results with the present study might be due to the difference in the user data-gathering tools and sample size. Also, cultural and behavioral differences might be effective on the sexual functionality of the couples.

No significant relation was observed between the perceived social support and adherence to treatment with the infertility factor in the present study. In line with the present study, no significant difference was observed between perceived social support and its aspects with infertility factors in some studies (26, 34). Desirable adherence to treatment in the present study despite its costs and problems might be influenced by the love for having a child and the importance of childbearing in Iranian culture (20).

It has been observed in many studies that the more social support an infertile woman receives from her family, spouse, and friends, the more favorable her sexual function will be and the better she will comply with infertility treatments. There was a linear positive relation between sexual function, perceived social support, and adherence to treatment in the studied infertile women in a way that improvement of perceived social support would lead to better sexual functionality and more adherence to treatment in infertile women. Some other studies have also reported similar results, revealing that improvement of perceived social support has caused better sexual functionality and even more adherence to treatment (13, 29, 33). In the conducted research, no studies were found with conflicting results and this indicates a significant relationship between these factors and also the importance of supportive factors and the effect of receiving support from the spouse, family, and friends on acceptance of and adherence to treatment in infertile women.

## 5. Conclusion

According to the results of the study, no significant relation was observed between infertility factors and sexual function, perceived social support, and adherence to treatment. However, if infertile women would receive appropriate social support, they would have better sexual functionality and more adherence to treatment. So, by providing appropriate education and counseling to the couples for the improvement of their perceived social support, their sexual functionality and adherence to treatment would also be improved and consequently, their infertility treatment would be completed.

##  Conflict of Interest

The authors declare that they have no conflict of interest.

## References

[B1] Deshpande PS, Gupta ASP (2019). Causes and prevalence of factors causing infertility in a public health facility. J Hum Reprod Sci.

[B2] Sun H, Gong T-T, Jiang Y-T, Zhang S, Zhao Y-H, Wu Q-J (2019). Global, regional, and national prevalence and disability-adjusted life-years for infertility in 195 countries and territories, 1990-2017: Results from a global burden of disease study, 2017. Aging (Albany NY).

[B3] De D, Mukhopadhyay P, Roy PK (2020). Experiences of infertile couples of West Bengal with male factor, female factor, and unexplained infertility factor: A qualitative study. J Psychosexual Health.

[B4] Doryanizadeh L, Morshed-Behbahani B, Parsanezhad ME, Dabbaghmanesh MH, Jokar A (2021). Calcitriol effect on outcomes of in vitro fertilization in infertile women with vitamin D deficiency: A double-blind randomized clinical trial. Z Geburtshilfe Neonatol.

[B5] Akhondi MM, Ranjbar F, Shirzad M, Behjati Ardakani Z, Kamali K, Mohammad K (2019). Practical difficulties in estimating the prevalence of primary infertility in Iran. Int J Fertil Steril.

[B6] Sheoran P, Sarin J (2015). Infertility in India: Social, religion and cultural influence. Int J Reprod Contracept Obstet Gynecol.

[B7] Mirzaei M, Namiranian N, Dehghani Firouzabadi R, Gholami S (2018). The prevalence of infertility in 20-49 years women in Yazd, 2014-2015: A cross-sectional study. Int J Reprod BioMed.

[B8] Mansouri M, Heydar Z, Lotfi R, Rahimzadeh Kiwi M, Tahamtan T (2021). [The relationship between sexual and marital satisfaction level and the causes of infertility in infertile couples who referred to Nahal infertility center in Alborz province]. AUMJ.

[B9] Karimi FZ, Taghipour A, Latifnejad Roudsari R, Kimiaee SA, Mazloum SR, Amirian M (2016). Psycho-social effects of male infertility in Iranian women: A qualitative study. Iran J Obstet Gynecol Infertil.

[B10] Bois K, Bergeron S, Rosen N, Mayrand M-H, Brassard A, Sadikaj G (2016). Intimacy, sexual satisfaction, and sexual distress in vulvodynia couples: An observational study. Health Psychol.

[B11] Karli P, Ozdemir AZ (2019). Sexual dysfunction in in-vitro fertilization (IVF) patients and the effect of ovarian reserve on sexual dysfunction. Ann Med Res.

[B12] Zare Z, Amirian M, Golmakani N, Mazlom R, Ahangar ML (2016). Sexual dysfunction in infertile women. Int J Reprod BioMed.

[B13] Sahraian K, Jafarzadeh F, Poursamar SL (2015). [The relationship between social support and marital satisfaction in infertile women based on infertility factor]. Nurs Midwif J.

[B14] Jafarkhany ZSM, Shabanian G, Manzary Tavakoly V (2017). [A comparison of marital adjustment, empathy and positive and negative emotions fertile and infertile women in Kerman]. J Woman Stud Family.

[B15] Maroufizadeh S, Karimi E, Vesali S, Omani Samani R (2015). Anxiety and depression after failure of assisted reproductive treatment among patients experiencing infertility. Int J Gynecol Obstet.

[B16] Hwu L-J, Hsu M-Y, Chuang H-L, Shih F-F, Lu Y-CA, Lee S-H (2022). Childbearing perceptions among Taiwanese women undergoing in vitro fertilization treatment: A qualitative study. J Transcult Nurs.

[B17] Gholamaliei B, Karimi-Shahanjarini A, Roshanaei G, Rezapour-Shahkolaei F (2016). [Medication adherence and its related factors in patients with type II diabetes]. J Educ Community Health.

[B18] Li S, He A, Yang J, Yin T, Xu W (2011). A logistic regression analysis of factors related to the treatment compliance of infertile patients with polycystic ovary syndrome. J Reprod Med.

[B19] Mahoney DE, Russell CL, Cheng A-L (2019). Medication adherence among women undergoing infertility treatment: A systematic review. Int J Women’s Health Reprod Sci.

[B20] Swift A, Reis P, Swanson M

[B21] Direkvand-Moghadam A, Delpisheh A, Direkvand-Moghadam A

[B22] Oindi FM, Murage A, Lema VM, Mukaindo AM (2019). Association of female sexual dysfunction and fertility: A cross sectional study. Fertil Res Pract.

[B23] Nazarpour S, Simbar M, Ramezani Tehrani F, Alavi Majd H (2015). Exercise and sexual dysfunction among postmenopausal women in Iran. Sci J School Public Health.

[B24] Cheraghi MA, Davari Dolatabadi E (2016). [Development and psychometric evaluation of the heart failure patients’ perceived social support inventory]. J Rafsanjan Univ Med Sci.

[B25] Bruwer B, Emsley R, Kidd M, Lochner C, Seedat S (2008). Psychometric properties of the multidimensional scale of perceived social support in youth. Compr Psychiatrry.

[B26] Ozturk A, Aba YA, Sik BA (2021). The relationship between stigma, perceived social support and depression in infertile Turkish women undergoing in vitro fertilization-embryo transfer. Arch Psychiatr Nurs.

[B27] Avşar B, Emul TG

[B28] Hays RD, Kravitz RL, Mazel RM, Sherbourne CD, DiMatteo MR, Rogers WH, et al (1994). The impact of patient adherence on health outcomes for patients with chronic disease in the medical outcomes study. J Behav Med.

[B29] Bashiri Nejadian A, Bayazi MH, Johari Fard R, Rajaei AR (2021). [The relationship between ambivalence over emotional expression and health-related quality of life and adherence to treatment in cancer patients: The mediating role of depression and social support: A descriptive study]. J Rafsanjan Univ Med Sci.

[B30] Moridi A, Roozbeh N, Yaghoobi H, Soltani Sh, Dashti S, Shahrahmani N, et al (2019). Etiology and risk factors associated with infertility. Int J Women’s Heal Reprod Sci.

[B31] Aramesh Sh, Diba E, Hassanzadeh S, Taghavi SA (2020). [Prevalence of infertility in Boyer-Ahmad city based on SIB system in 2016-2018: A cross sectional Study]. Armaghan Danesh.

[B32] Janati S, Poormoosavi SM, Tirkesh F (2019). [Survey of the causes of infertility in patients referred to Dezful infertility center from 1393 to 1396]. Jundishapur Sci Med J.

[B33] Besharat MA, Lashkari M, Rezazadeh MR (2015). [Explaining adjustment to infertility according to relationship quality, couples' beliefs and social support]. J Family Psychol.

[B34] Ataman H, Aygun O, Pekcan N, Doğan Merih Y (2021). The relationship between the perceived social support levels and levels of adjustment to the infertility problem of women who received infertility treatment. Izmir Democracy Univ Health Sci J.

[B35] Baghiani Moghadam MH, Aminian AH, Abdoli AM, Seighal N, Falahzadeh H, Ghasemi N (2011). Evaluation of the general health of the infertile couples. Iran J Reprod Med.

[B36] Diamond MP, Legro RS, Coutifaris C, Alvero R, Robinson RD, Casson PA, et al (2017). Sexual function in infertile women with polycystic ovary syndrome and unexplained infertility. Am J Obstet Gynecol.

